# Genetic Architecture of Natural Variation in Rice Nonphotochemical Quenching Capacity Revealed by Genome-Wide Association Study

**DOI:** 10.3389/fpls.2017.01773

**Published:** 2017-10-13

**Authors:** Quanxiu Wang, Hu Zhao, Junpeng Jiang, Jiuyue Xu, Weibo Xie, Xiangkui Fu, Chang Liu, Yuqing He, Gongwei Wang

**Affiliations:** National Key Laboratory of Crop Genetic Improvement, National Center of Plant Gene Research (Wuhan), Huazhong Agricultural University, Wuhan, China

**Keywords:** rice, nonphotochemical quenching, genome-wide association study, QTLs, *OsPsbS1*

## Abstract

The photoprotective processes conferred by nonphotochemical quenching (NPQ) serve fundamental roles in maintaining plant fitness and sustainable yield. So far, few loci have been reported to be involved in natural variation of NPQ capacity in rice (*Oryza sativa*), and the extents of variation explored are very limited. Here we conducted a genome-wide association study (GWAS) for NPQ capacity using a diverse worldwide collection of 529 *O. sativa* accessions. A total of 33 significant association loci were identified. To check the validity of the GWAS signals, three F2 mapping populations with parents selected from the association panel were constructed and assayed. All QTLs detected in mapping populations could correspond to at least one GWAS signal, indicating the GWAS results were quite reliable. *OsPsbS1* was repeatedly detected and explained more than 40% of the variation in the whole association population in two years, and demonstrated to be a common major QTL in all three mapping populations derived from inter-group crosses. We revealed 43 single nucleotide polymorphisms (SNPs) and 7 insertions and deletions (InDels) within a 6,997-bp DNA fragment of *OsPsbS1*, but found no non-synonymous SNPs or InDels in the coding region, indicating the PsbS1 protein sequence is highly conserved. Haplotypes with the 2,674-bp insertion in the promoter region exhibited significantly higher NPQ values and higher expression levels of *OsPsbS1*. The *OsPsbS1* RNAi plants and CRISPR/Cas9 mutants exhibited drastically decreased NPQ values. *OsPsbS1* had specific and high-level expression in green tissues of rice. However, we didn't find significant function for *OsPsbS2*, the other rice *PsbS* homologue. Manipulation of the significant loci or candidate genes identified may enhance photoprotection and improve photosynthesis and yield in rice.

## Introduction

Although light is necessary for plants to drive photosynthesis, absorption of excess light by pigment molecules can cause severe photo-oxidative damage and inhibit photosynthesis. Most plants receive more sunlight than they can actually use for photochemistry reaction on a daily as well as seasonal basis (Murchie and Niyogi, 2011). In addition, light in plant canopies is very dynamic, and the levels of absorbed irradiance in leaves under field conditions can vary over several orders of magnitude in seconds (Külheim et al., 2002). Thus, to optimize photosynthesis and growth, plants have evolved a variety of photoprotective and photoacclimatory mechanisms that operate at different time scales (Li et al., 2009). For example, photoreceptors such as phototropin, neochrome, and cryptochrome can sense excess light and relay signals for chloroplast avoidance movement and gene expression responses. Excess excitation energy in the photosystem II (PSII) antenna complex can also be harmlessly dissipated as heat, which is observable as a process named nonphotochemical quenching of chlorophyll fluorescence (Müller et al., 2001; Li et al., 2002).

Nonphotochemical quenching (NPQ) can be divided into multiple components according to their relaxation kinetics in darkness following a period of illumination (Müller et al., 2001; Nilkens et al., 2010; Ruban, 2016). The major and most rapid component in plants is the pH- or energy-dependent component, qE. The qE-type of NPQ or feedback de-excitation, is a major photoprotective strategy that operates on a timescale of seconds to minutes and involves a regulated thermal dissipation of excess absorbed light energy. The rapid induction and relaxation of qE are required to cope with frequent, rapid and irregular changes in the natural light environment (Külheim et al., 2002). A current model of qE in plants is as follows. When light absorption exceeds the capacity for light utilization in assimilatory reactions, a decrease in the proton conductance of the chloroplast ATPase results in a rapid decrease in thylakoid lumen pH. The low thylakoid lumen pH induces de-epoxidation of violaxanthin to antheraxanthin and zeaxanthin via the xanthophyll cycle and protonation of a photosystem II protein, PsbS. Together, binding of protons and xanthophylls (zeaxanthin and lutein) to specific sites in the PSII antenna causes a conformational change that switches into a quenched state (Müller et al., 2001; Bianchi et al., 2010; Ruban, 2016).

Natural variation in NPQ capacity has been reported in different plant species. Jung and Niyogi (2009) surveyed the genetic basis for natural variation of NPQ among Arabidopsis accessions. In spite of significant differences in NPQ, previously identified NPQ factors such as xanthophyll cycle de-epoxidation state and the amount of the PsbS protein were indistinguishable between the high and the low NPQ accessions, suggesting that the differences in NPQ variation in Arabidopsis may be controlled by new genes. Using a F2 mapping population, two high NPQ QTLs were identified and one was validated by the phenotype of near isogenic lines (Jung and Niyogi, 2009). In rice, Kasajima et al. (2011) identified significant differences in qE capacity between *indica* and *japonica* cultivars. Using backcrossed inbred lines derived from an inter-subspecific cross, two QTLs on chromosome 1 were identified for the magnitude of NPQ, with one harboring the *OsPsbS1* gene. Comparison of the public available genomic sequence around *OsPsbS1* revealed that a 2.7-kb region upstream of the translation initiation site of *OsPsbS1* was lost in 9311 (*indica*) compared with Nipponbare (*japonica*). Existence of this deletion was also analyzed by PCR markers in several *japonica* and *indica* cultivars, and this deletion was found to be correlated with NPQ capacity (Kasajima et al., 2011). Nuruzzaman et al. (2014) further demonstrated the 2.7-kb insertion fragment in Nipponbare contains a Mutator-like-element (MULE). However, the nucleotide diversity, haplotype effects, and expression patterns of *OsPsbS1* haven't been characterized in detail.

As the staple food for more than half of the world's population, rice is normally cultivated under high natural illumination in summer season, and light may be frequently in excess of that required for CO_2_ assimilation. The ability of qE to safely dissipate excess excitation energy is thus very important for plant fitness and sustainable yield in rice. Although *OsPsbS1* has been identified (Kasajima et al., 2011), the extent of variations in NPQ capacity has not been fully explored due to the very limited number of rice accessions used previously. Compared with QTL linkage mapping approach, genome-wide association study (GWAS) can greatly increase the range of natural variation and the number of significant loci, especially for complex traits (Huang et al., 2010; Zhao et al., 2011). In this study, we conducted a GWAS for NPQ capacity using a diverse worldwide collection of 529 *O. sativa* accessions. Thirty-three significant association regions were identified, and three F2 mapping populations with parents selected from the association panel were tested for validation of significant GWAS signals. *OsPsbS1* was found to be a major determinant for natural variation of NPQ capacity. To our knowledge, this study provides the most comprehensive investigation into the genetic architecture of natural variation of NPQ capacity in rice to date. The nucleotide diversity, haplotype effects, and expression patterns of *OsPsbS1* were further characterized. The function of *OsPsbS1* was verified by RNA interference and CRISPR/Cas9 knockout mutants. The implications of the results were discussed.

## Materials and methods

### Plant materials for association analysis

The association panel consisted of a diverse collection of 529 *O. sativa* accessions including both landraces and elite varieties. The details about the accessions, including accession name, country of origin, longitude and latitude origin, and subpopulation identity, have been reported previously (Wang et al., 2015; Xie et al., 2015) and are available at the RiceVarMap (http://ricevarmap.ncpgr.cn).

### Genome-wide association analyses

The sequencing, SNP identification, and imputation of the association panel have been reported in previous studies (Wang et al., 2015; Xie et al., 2015). The SNPs of the 529 *O. sativa* accessions are available at the RiceVarMap (http://ricevarmap.ncpgr.cn). There were 2,767,191, 1,857,866, 1,925,362, and 3,916,415 SNPs (minor allele frequency ≥0.05; the number of accessions with minor alleles ≥6) used in GWAS for groups of *Indica, Japonica, Aus*, and the whole panel, respectively. We performed GWAS using the linear mixed model (LMM) and the simple linear regression model (LR) provided by FaST-LMM program (Lippert et al., 2011). The population structure of Q matrix and kinship (K matrix) was taken into account as cofactor when performing association mapping using the LMM method. Using a method described by Li et al. (2012), the effective numbers of independent SNPs and suggestive thresholds were calculated. The effective numbers of independent SNPs were 571,843, 245,348, 235,880, and 757,578 for groups *Indica, Japonica, Aus*, and the whole panel, respectively. The suggestive *P* values set as thresholds to identify significant association signals by LMM were 1.75 × 10^−6^, 4.08 × 10^−6^, 4.24 × 10^−6^, and 1.32 × 10^−6^ for groups *Indica, Japonica, Aus*, and the whole panel, respectively. To obtain independent association signals, multiple SNPs exceeding the threshold in a 5 Mb region were clustered by *r*^2^ of LD ≥0.25 and SNPs with the minimum *P*-value in a cluster were considered as lead SNPs.

### Field experiments and evaluation of NPQ values in the association panel

Field trials were conducted in two years. The rice seeds were sown in the Experimental Station of Huazhong Agricultural University, Wuhan, China, in mid-May of 2012 and 2013. Seedlings about 25 days old were transplanted to the field. Each plot consisted of 4 rows with 10 plants each. The planting density was 16.5 cm between plants in a row, and the rows were 26 cm apart. Field management, including irrigation, fertilizer application and pest control, followed essentially the normal agricultural practice.

Three plants in the middle from the third row of each accession were subject to investigation for NPQ values before heading stage. Flag leaves were excised and floated on ion-exchanged water containing 0.01% Triton X-100 or 0.1% agar in centrifuge tubes. Leaves were dark-acclimated for at least 2 h. Chlorophyll fluorescence was measured with the PAM-2500 portable chlorophyll fluorometer (WALZ, Germany). Actinic lights were supplemented at a PPFD of 1,000 μmol m^−2^ s^−1^ for 5 min before measurements of Fm' to calculate parameter NPQ. The Stern-Volmer type nonphotochemical fluorescence quenching (Bilger and Björkman, 1990) was calculated by the following equation: NPQ = Fm/Fm'-1.

### QTL analysis

Three F2 populations were constructed for QTL mapping, with parents selected from the association panel. All of them were derived from inter-group crosses, an *IndI* by *TrJ* cross (Sadu-cho/Dourado Precoce), an *IndI* by *TeJ* cross (Zegu/Weiguo), and an *Aus* by *TeJ* cross (Dular/Akitakomachi). For each F2 population, 144 individuals were investigated for genotypic and phenotypic assay. Genotypic assay was performed using SSR markers, and measurement of NPQ values was conducted with the same method as described above. QTL analysis was conducted using composite interval mapping in QTL Cartographer (http://statgen.ncsu.edu/qtlcart/cartographer.html). The SSR markers used in the QTL analysis are presented in Table S4.

### Nucleotide diversity analysis

The genomic DNA extraction and sequencing were carried out according to the methods described by Du et al. (2011). The DNA sequences were assembled using SeqMan software and all polymorphisms were confirmed visually to ensure the accuracy. Sequences were inspected initially using Sequencher V5.4 program and edited manually using the SeqMan software. The genomic sequences were aligned by MAGE5. Nucleotide diversity and Tajima's *D* statistics were calculated using the DnaSP 5.0 program.

### Quantitative RT-PCR analysis

Total RNA was isolated from rice leaves using TRIzol Reagent (TransGen) according to the manufacturer's instructions. Approximately 3 μg of RNA sample was subjected to RNase-free DNaseI (Invitrogen) treatment and reverse transcribed using M-MLV Reverse Transcriptase (Invitrogen) with Oligo(dT)15. Quantitative RT–PCR was performed in a ViiA 7 Real-Time PCR system (Applied Biosystems) using FastStart Universal SYBR Green Master (Rox) superMIX (Roche). *Ubiquitin* was used as a reference gene in the qRT–PCR experiments. The measurements were obtained using the relative quantification method. The significant difference was analyzed statistically by student's t test or one-way analysis of variance (ANOVA). Primer pairs for qRT-PCR analysis are listed in Table S3.

### Vector construction for RNAi knockdown and CRISPR/Cas9 knockout

To generate the RNAi constructs, the 425-bp fragment from the exon of *OsPsbS1* was amplified by PCR using specific primers (Table S3), and cloned into the *Kpn*I-*Bam*HI and *Spe*I-*Sac*I restriction sites of RNAi construct ds1301.

The CRISPR/Cas9 vector system for multiplex targeting of gene sites in monocot plants was kindly provided by Professor Yaoguang Liu (South China Agriculture University). In order to increase the efficiency of the targeting, we designed two target special sequences for each gene in exon regions. Briefly, the target site sequences were cloned into the sgRNA expression cassette which contains rice promoter, and then cloned into the *Bsa*I restriction sites of vector pYLCRISPR/Cas9-MH. The primers used in constructing the sgRNA vectors for *OsPsbS1* and *OsPsbS2* are listed in Table S3.

The RNAi and CRISPR/Cas9 constructs were independently introduced into *Agrobacterium tumefaciens* strain EHA105, and then transformed into Zhonghua 11.

### Histochemical staining of GUS activity

Histochemical staining of GUS activity in rice tissues was conducted essentially as described previously. Various tissues of transgenic-positive transformants (root, leaf, sheath, panicle, stem and mature seed) were incubated in GUS staining solution (50 mM sodium phosphate at pH 7.0, 10 mM Na_2_-EDTA, 0.1% Triton X-100, 1 mg/ml X-Gluc, 100 μg/ml chloramphenicol, 1 mM potassium ferricyanide, 1 mM potassium ferrocyanide, and 20% methanol) at 37°C for 2–10 h after 15-min vacuum filtration. After GUS staining, the samples were incubated in 70% ethanol to remove chlorophyll and photographs were taken under a dissecting microscope (Leica MZFLIII).

## Results

### Phenotypic variation of NPQ in the association panel

As described by us previously (Wang et al., 2015; Xie et al., 2015), the whole association panel exhibited a distinct population structure, and was classified into 98 *indica I* (*IndI*), 105 *indica II* (*IndII*), 92 *indica* intermediate, 91 temperate *japonica* (*TeJ*), 44 tropical *japonica* (*TrJ*), 21 *japonica* intermediate, 46 *Aus*, and 14 *VI* (an intermediate group). The basic information of 529 accessions is available at the RiceVarMap (http://ricevarmap.ncpgr.cn).

The mean values and standard deviation of NPQ in different groups and all accessions in 2012 and 2013 (designated as NPQ_12 and NPQ_13, respectively) are presented in Table 1. A significant correlation was observed between NPQ_12 and NPQ_13 in the whole association population (*r* = 0.64). Large phenotypic variation was mainly detected between different subspecies in both years. Comparisons of the trait values between *indica* (consisting of *IndI, IndII* and *indica* intermediate) and *japonica* (consisting of *TeJ, TrJ* and *japonica* intermediate) revealed that *japonica* generally had significantly higher values in NPQ_12 (*P* = 2.79 × 10^−47^) and NPQ_13 (*P* = 2.47 × 10^−59^). *Aus* exhibited similar magnitude of NPQ values to *indica* (*P* > 0.05) in both two years, while much lower values than that in *japonica* (*P* = 7.68 × 10^−11^ for NPQ_12 and *P* = 1.29 × 10^−17^ for NPQ_13). However, no statistically significant differences in NPQ values were observed between *IndI* and *IndII* or between *TeJ* and *TrJ*, with the only exception occurred between *IndI* and *IndII* in NPQ_13 (*P* = 0.00028).

**Table 1:** The means and standard deviation of NPQ values in different groups and the whole panel in 2012 and 2013.

**Group**	**NPQ_12**		**NPQ_13**	
	**Means**	**SD**	**Means**	**SD**
*IndI*	2.371	0.209	2.450	0.247
*IndII*	2.366	0.175	2.564	0.165
*TeJ*	2.740	0.230	2.941	0.208
*TrJ*	2.721	0.279	2.980	0.296
*Aus*	2.421	0.213	2.567	0.206
*Ind_All*	2.363	0.191	2.522	0.211
*Jap_All*	2.716	0.256	2.946	0.245
All accessions	2.487	0.273	2.665	0.295

### Identification of significant loci for NPQ values through GWAS

**Figure 1:**
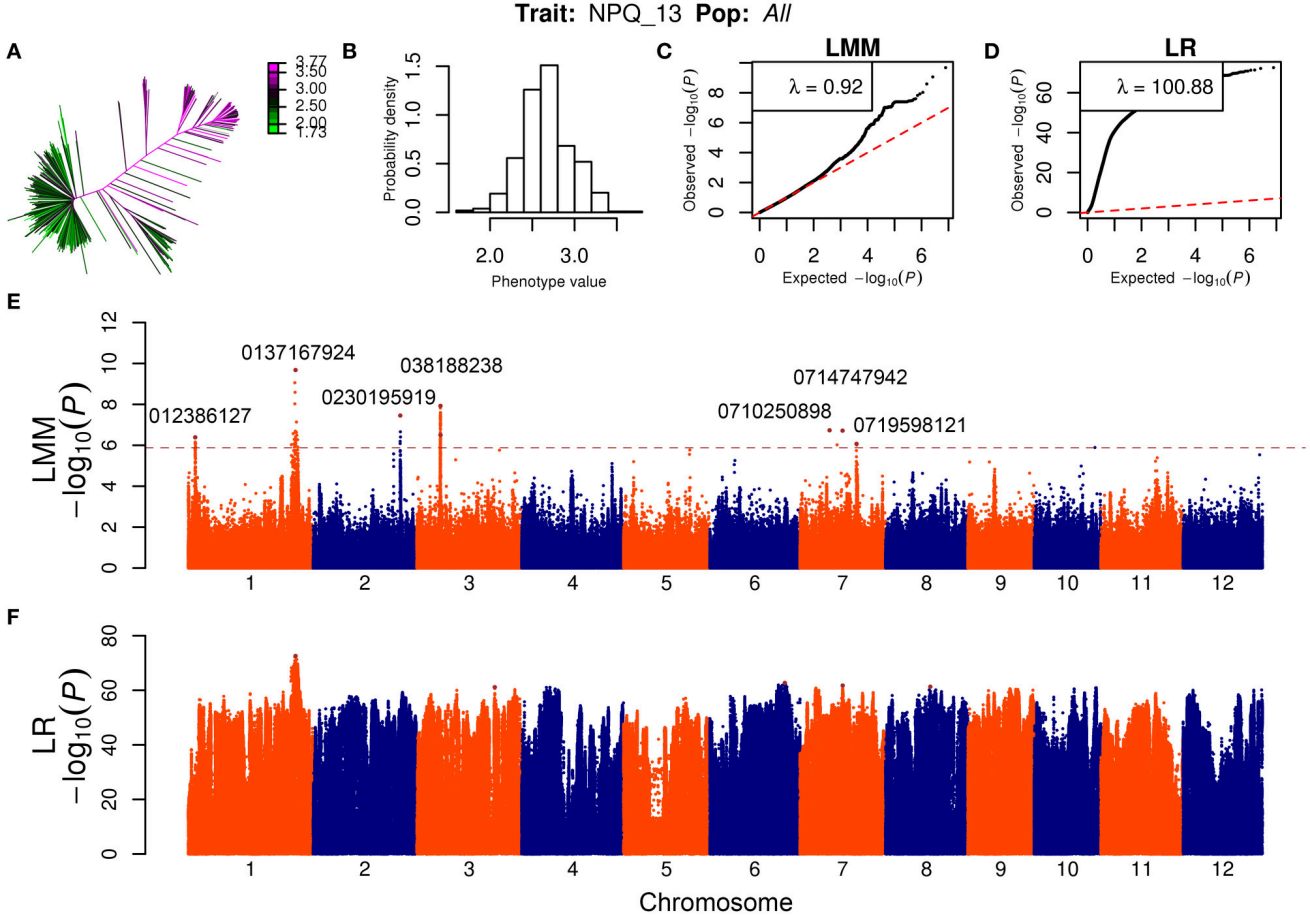
GWAS of NPQ values in 2013 for the whole association population. **(A,B)** The heatmap **(A)** and histogram **(B)** distribution of NPQ values in 2013 in 529 accessions. **(C,D)** Q-Q plot of the expected null distribution and the observed *P*-value using the linear mixed model (LMM) **(C)** and the simple linear regression (LR) model **(D)**. **(E,F)** Genome-wide *P*-values for the LMM **(E)** and simple LR model **(F)**. The horizontal dashed line indicates the significance thresholds set as *P* = 1.32 × 10^−6^ for the whole population by LMM. The SNP positions of representative peak signals in LMM were denoted.

Genome-wide association analyses were performed separately in the whole population and in the *indica, japonica*, and *aus* subpopulations for each year. A total of 33 significant association regions were identified at linear mixed models (LMM) (lead SNPs less than or around 300 kb were considered as caused by one common gene and counted as only one association region) for NPQ values. The details about these significant association signals are listed in Table 2. The quantile-quantile plots and Manhattan plots of LMM for NPQ_13 in the whole population are illustrated in Figure 1 as an example.

**Table 2:** The details about the significant GWAS signals for NPQ values and candidate genes.

**Chr**	**SNP pos (bp)**	***P*_LMM_**	***q^2^***	**Group**	**Trait**	**Detected in different groups**	**Detected in different years**	**Known genes or candidate genes for reference**	**Annotation for candidate genes**
1	129,845	^*^7.7e–07	0.181	*Ind_All*	NPQ_13			LOC_Os01g01340	light-induced protein 1-like, putative, expressed
1	2,386,127	^*^4.1e–07	0.0005	*All*	NPQ_13			LOC_Os01g05080	thylakoid lumenal protein, putative, expressed
1	21,398,043	^*^1.1e–06	0.0555	*Ind_All*	NPQ_13				
1	37,090,439	^*^5.4e–10	0.4007	*All*	NPQ_12		yes	*OsPsbS1*	
1	37,167,924	^*^2.1e–10	0.4677	*All*	NPQ_13		yes	*OsPsbS1*	
2	17,475,225	^*^6.9e–07	0.0995	*Ind_All*	NPQ_12				
2	30,195,919	^*^3.5e–08	0.0134	*All*	NPQ_13			LOC_Os02g49350	plastocyanin-like domain containing protein, putative, expressed
3	3,147,551	^*^1.2e–07	0.0946	*Ind_All*	NPQ_12				
3	8,146,159	^*^2.4e–06	0.1821	*Jap_All*	NPQ_13	yes			
3	8,188,238	^*^1.2e–08	0.2832	*All*	NPQ_13	yes			
3	8,825,931	^*^3.7e–06	0.2057	*Jap_All*	NPQ_13			LOC_Os03g15810	AAA-type ATPase family protein, putative, expressed
3	15,656,168	^*^1.1e–06	0.098	*All*	NPQ_12				
3	33,364,634	^*^3e–08	0.2228	*All*	NPQ_12				
4	3,839,288	^*^4.9e–08	0.0184	*All*	NPQ_12				
4	4,383,955	^*^3.7e–06	0.397	*Aus*	NPQ_13				
4	5,383,505	^*^1e–06	0.0718	*Ind_All*	NPQ_12				
5	844,178	^*^1e–07	0.0261	*All*	NPQ_12				
6	29,050,672	^*^5.4e–08	0.1791	*Ind_All*	NPQ_13				
7	2,508,598	^*^1.3e–06	0.3757	*All*	NPQ_12			LOC_Os07g05360	photosystem II 10 kDa polypeptide, chloroplast precursor, putative, expressed
7	4,380,583	^*^1.1e–06	0.1646	*Ind_All*	NPQ_13				
7	9,172,004	^*^1.9e–11	0.2327	*Ind_All*	NPQ_13	yes	yes	LOC_Os07g15770 (*Ghd7*)	CCT motif family protein
7	9,162,584	^*^3.2e–07	0.2145	*All*	NPQ_12	yes	yes	LOC_Os07g15770 (*Ghd7*)	CCT motif family protein
7	10,250,898	^*^1.9e–07	0.1405	*All*	NPQ_13				
7	12,842,943	^*^1.9e–09	0.2361	*Ind_All*	NPQ_13				
7	14,747,942	^*^1.9e–07	0.1364	*All*	NPQ_13	yes			
7	14,747,942	^*^9.6e–10	0.2369	*Ind_All*	NPQ_13	yes			
7	16,028,272	^*^7.5e–07	0.1903	*Ind_All*	NPQ_13				
7	19,598,121	^*^8.5e–07	0.1631	*All*	NPQ_13			LOC_Os07g32880	ATP synthase gamma chain, putative, expressed
7	27,408,489	^*^6.2e–07	0.4935	*Aus*	NPQ_13				
8	24,543,491	^*^1.3e–06	0.1392	*All*	NPQ_12				
9	9,738,425	^*^6.9e–07	0.5086	*Aus*	NPQ_13				
9	15,814,138	^*^4.1e–06	0.1834	*Jap_All*	NPQ_12			LOC_Os09g26260	AAA-type ATPase family protein, putative, expressed
10	958,084	^*^1e–06	0.2265	*Jap_All*	NPQ_13				
10	14,342,784	^*^2.3e–06	0.1947	*Jap_All*	NPQ_13			LOC_Os10g27220	phospholipid-transporting ATPase 3, putative, expressed
10	16,283,435	^*^2.6e–07	0.0868	*Ind_All*	NPQ_12				
12	10,871,643	^*^3.1e–06	0.39	*Aus*	NPQ_13				
12	21,054,623	^*^2.2e–06	0.0514	*Jap_All*	NPQ_13				

Chr, chromosome; SNP Pos, SNP position on rice genome assembly MSU version v6.1; P_LMM_, P value from LMM; q^2^, variance explained by the SNP effect;

* *indicates P value from LMM was lower than the significance thresholds*.

GWAS results over the two years were compared. Among them, two regions were commonly detected in both NPQ_12 and NPQ_13. Significant genetic heterogeneity was observed when comparing GWAS results performed in the different groups and in the whole population. Only three significant association regions were detected in at least two different groups (Table 2).

To search for candidate genes, the significant GWAS signals were compared with the positions of known or putative genes involved in NPQ regulation. We found that *OsPsbS1* corresponds to one significant association region that was repeatedly detected and explained more than 40% of the variation in the whole population in both 2012 and 2013 (Table 2). However, no other previously identified NPQ key factors, such as genes related to xanthophyll cycle and lutein synthesis (Niyogi et al., 1998, 2001; Ruban, 2016), were found to be close to the detected significant association signals. It suggests the NPQ variation in rice may be controlled by a lot of new genes. Close to the GWAS peak signals, we found some candidate genes encoding light-induced protein 1-like, thylakoid lumenal protein, photosystem II (PSII) 10 kDa polypeptide, ATPase, ATP synthase gamma chain, and plastocyanin-like domain containing protein (Table 2). These candidate genes might be involved in processes of electron transport, buildup of proton gradient, PSII light harvesting antenna structure and light harvesting complex II (LHCII) rearrangements that trigger and regulate NPQ scenario (Ruban, 2016).

### Validation of GWAS signals with QTL mapping

To investigate whether GWAS significant signals could be detected in bi-parental QTL mapping, we constructed three F2 mapping populations derived from inter-group crosses, with parents selected from the association panel. In the F2 population of *IndI* by *TrJ* (Sadu-cho/Dourado Precoce), one major QTL qSD1.1, explaining 29.4% of the phenotypic variation, located in the interval between RM212 and RM6504 on chromosome 1, was identified. In addition, a QTL qSD8.1, located in the interval between RM556 and RM80 on chromosome 8, also showed significant effects on NPQ values. The detailed information about these significant QTLs is presented in Table 3.

**Table 3:** Summary of significant QTLs for NPQ values identified in three F2 populations of this study.

**Cross of F_2_ population**	**QTL**	**Chr**	**Interval**	**LOD**	**Add^a^**	**Dom^b^**	**Var (%)^c^**	**Corresponding lead SNP position in GWAS or known genes**
Sadu-cho/Dourado Precoce (*IndI* ^*^TrJ)	qSD1.1	1	RM212-RM6504	10.1	−0.1724	0.0279	29.4	*OsPsbS1*
	qSD8.1	8	RM556-RM80	2.7	0.086	0.0282	5.4	24,543,491
Zegu/Weiguo (*IndI* ^*^TeJ)	qZW1.1	1	RM8278-RM315	12.6	−0.1793	0.0253	33.6	*OsPsbS1*
	qZW7.1	7	RM7571-RM13.40	2.7	−0.1369	0.0371	11.1	12,842,943
Dular/Akitakomachi (*Aus* ^*^TeJ)	qDA1.1	1	RM212-RM315	13.7	−0.1857	0.0212	37.0	*OsPsbS1*
	qDA7.1	7	RM6344-RM420	3.4	−0.0771	0.0092	6.0	27,408,489
	qDA9.1	9	RM5657-RM434	3.4	−0.0764	0.0396	9.1	15,814,138

a *Additive effects. Positive values indicate that the allele from Sadu-cho or Zegu or Dular can increase the phenotypic value, whereas negative values indicate the allele from the counterpart parent Dourado Precoce or Weiguo or Akitakomachi can increase the phenotypic value*.

b *Dominance effect. Positive values imply that the heterozygotes had higher phenotypic value than the means of the two homozygotes, whereas negative values imply that the heterozygotes had lower phenotypic value than the means of the two homozygotes*.

c *Variance explained*.

In the *IndI* by *TeJ* (Zegu/Weiguo) F2 population, similarly, one major QTL qZW1.1, explaining 33.6% of the phenotypic variation, located in the interval between RM8278 and RM315 on chromosome 1, was detected. Furthermore, a QTL qZW7.1, located in the interval between RM7571 and RM13.40 on chromosome 7, exhibited significant effects (Table 3).

In the F2 population of *Aus* by *TeJ* (Dular/Akitakomachi), one major QTL qDA1.1, explaining 37.0% of the phenotypic variation, located in the interval between RM212 and RM315 on chromosome 1, was also identified. Additionally, two QTLs qDA7.1 (located in the interval between RM6344 and RM420 on chromosome 7) and qDA9.1 (located in the interval between RM5657 and RM434 on chromosome 9), showed significant effects on NPQ values (Table 3).

After comparing the regions of these QTLs with GWAS signals, we found that all the major QTL detected on chromosome 1 corresponds to *OsPsbS1*, and other significant QTLs also co-localize with at least one GWAS signal (Table 3), indicating the GWAS results were quite reliable.

### Characterization of *OsPsbS1*, a major locus for natural variation of NPQ capacity in rice

To characterize the nucleotide diversity of *OsPsbS1*, the whole genomic DNA sequences around *OsPsbS1* from 480 accessions in the association panel were sequenced and analyzed. In total, 43 SNPs and 7 insertions and deletions (InDels) were detected in the aligned 6,997 basepairs, which include 4,022-bp promoter and 2975-bp coding regions (Table 4). Varied DNA polymorphisms were observed in different regions of the *OsPsbS1* genome. We found no nucleotide polymorphic sites in exon-1, intron-1, and exon-2, and only one synonymous SNP in exon-3, indicating the structure and function of PsbS protein are highly conserved in rice. In the whole germplasm population, the pairwise nucleotide diversity parameter (π) and the level of the Watterson estimator (θ) in intron-2 and 3′UTR regions were much higher than that in the exons, promoter, and 5′UTR regions. Tajima's *D* values reached a significant positive level in the *OsPsbS1* gene region (Table 4). Considering the strong population stratification, we also tested these parameters within the two subpopulations (*indica* and *japonica* group). The sequence diversities of *indica* in intron-2 and 3′UTR regions were also much higher than that in the exons, promoter, and 5′UTR regions. Compared with *indica*, lower levels of nucleotide diversity were observed in *japonica*. The Tajima's *D* values showed negative but not statistically significant values in both *indica* and *japonica* group (Table 4).

**Table 4:** Summary of DNA polymorphic sites of *OsPsbS1* genome.

**Parameter**	**Promoter**	**Gene region**	**5′UTR**	**Exon-1**	**Intron-1**	**Exon-2**	**Intron-2**	**Exon-3**	**3′UTR**
Length, bp	4,022	2,975	331	198	116	117	1,379	492	342
SNP sites	15	28	2	0	0	0	18	1	7
Indels	2	5	0	0	0	0	4	0	1
**WHOLE POPULATION**									
π	0.00224	0.00306	0.00283	0	0	0	0.00444	0.00024	0.00581
θ	0.00165	0.00145	0.00090	0	0	0	0.00205	0.00030	0.00318
Tajima's *D*	0.82255	2.89836^**^	2.62209^*^	–	–	–	2.86141^**^	−0.18476	1.58889
***japonica*** **GROUP**									
π	0.00045	0.00033	0.00031	0	0	0	0.00050	0	0.00055
θ	0.00146	0.00097	0.00108	0	0	0	0.00143	0	0.00164
Tajima's *D*	−1.71154	−1.75267	−1.01483	–	–	–	−1.60050	–	−1.11096
***indica*** **GROUP**									
π	0.00058	0.00131	0.00014	0	0	0	0.00242	0	0.00149
θ	0.00109	0.00137	0.00098	0	0	0	0.00212	0	0.00339
Tajima's *D*	−1.02660	−0.13007	−1.12832	–	–	–	0.35855	–	−0.74251

*π, Average number of nucleotide differences per site between two sequences; θ, Watterson estimator; Tajima's D, test for neutral selection*.

* Significant at P < 0.05;

** *significant at P < 0.01*.

**Figure 2:**
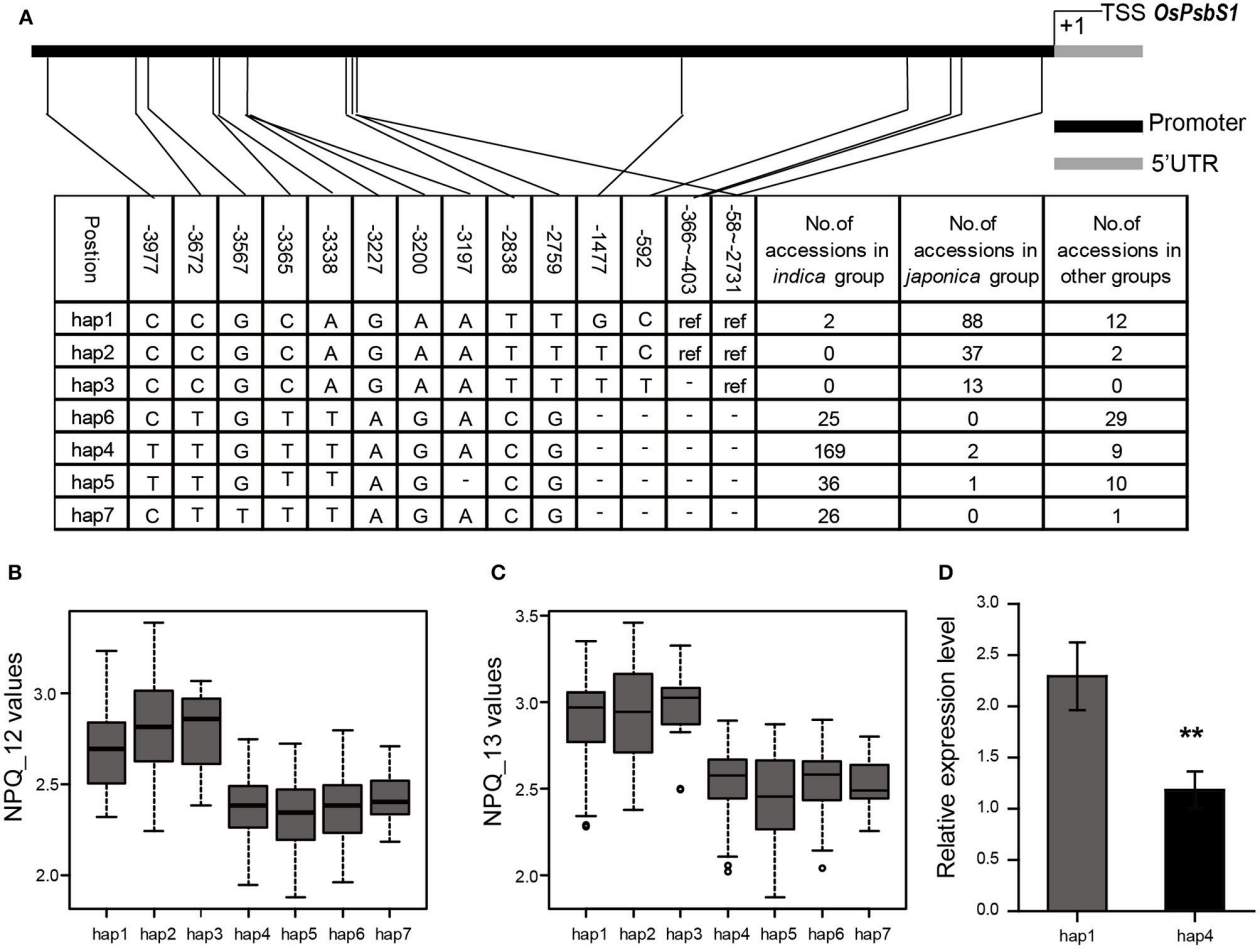
Haplotype analysis of the *OsPsbS1* promoter region in the 480 accessions. **(A)** Seven haplotypes (hap1-hap7) were detected. The 4,022-bp promoter region is shown in graphics on the top. The position of every SNP is shown in the first row. The number of accessions in each haplotype is shown in the right columns. Ref, sequence identical to Nipponbare; –, sequence deleted. **(B)** The distribution of NPQ_12 values in the seven haplotypes. **(C)** The distribution of NPQ_13 values in the seven haplotypes. **(D)** The transcript levels of *OsPsbS1* in flag leaves of hap1 and hap4 accessions. ^**^The differences in expression levels between hap1 and hap4 are significant at *P* < 0.01.

The sequencing results showed that the vast majority of *japonica* varieties has the 2,674-bp insertion fragment in the promoter region of *OsPsbS1*, compared with most *indica* and *aus* varieties. We further analyzed the effects of haplotypes based on 15 SNPs and 2 indels in the promoter region. For the strictness of statistical analysis, we only focused on those haplotypes shared by at least five accessions. The 480 accessions were accordingly divided into 7 haplotypes, among which hap1-3 contained the 2674-bp insertion, whereas hap4-7 didn't (Figure 2A). It was found that haplotypes with the 2674-bp insertion exhibited significantly higher values in NPQ_12 (Figure 2B) and NPQ_13 (Figure 2C) than other haplotypes without the insertion. We also compared the expression levels of *OsPsbS1* in flag leaves of 49 randomly chosen accessions with the two major haplotypes, hap1 and hap4. Real-time PCR results showed that the expression level of *OsPsbS1* in hap1 was much higher than that in hap4 (Figure 2D). The results suggest that variation of the *OsPsbS1* expression levels might be the main cause of natural variation of NPQ capacity in the whole association panel.

**Figure 3:**
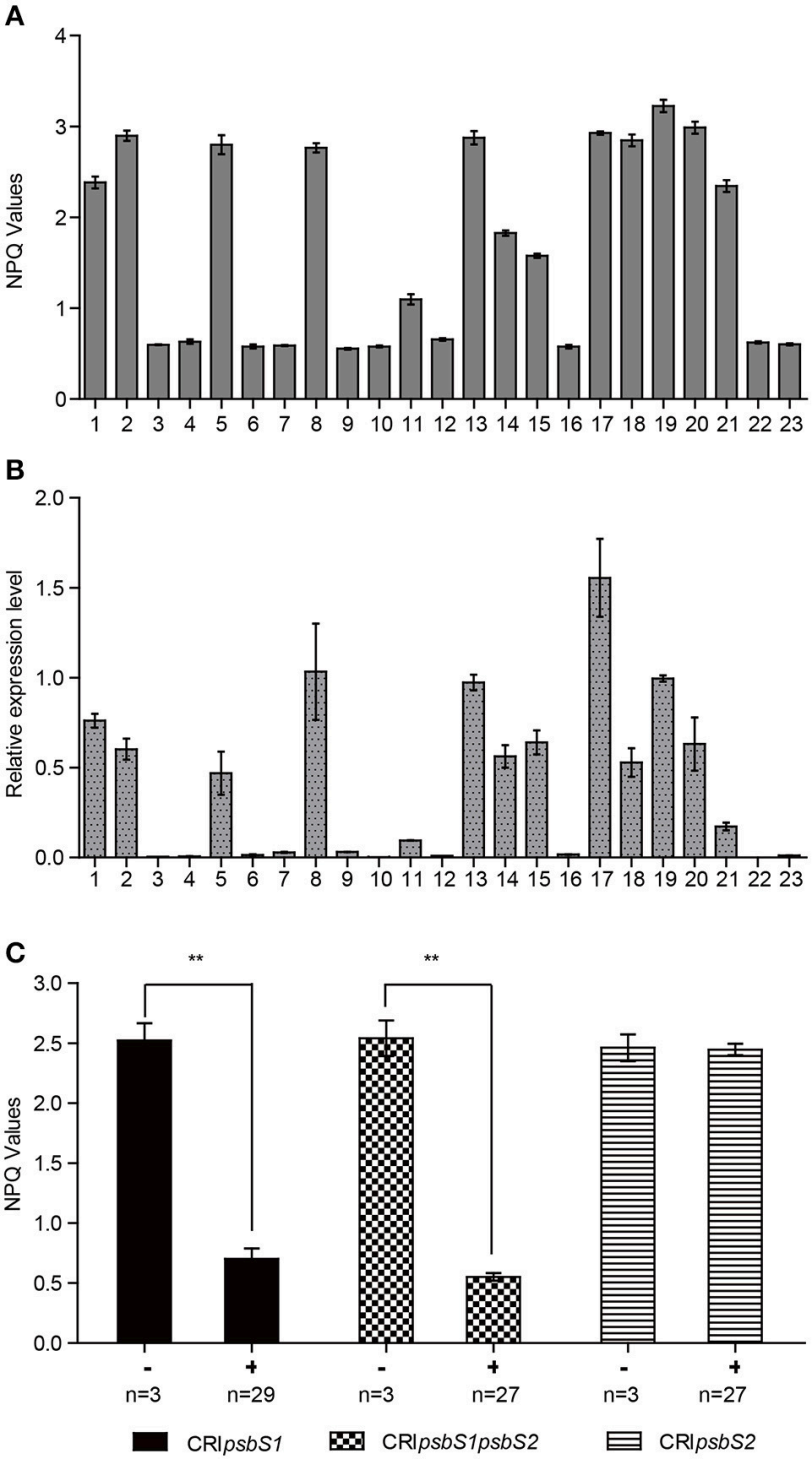
The NPQ values in *OsPsbS1* RNAi and CRISPR mutant plants. **(A,B)** The expression levels of *OsPsbS1* **(A)** and corresponding NPQ values **(B)** in flag leaves of *OsPsbS1* RNAi plants. **(C)** The NPQ values in CRISPR mutants targeting the single gene *OsPsbS1* or *OsPsbS2* and both of the two genes. The number of plants *(n)* were shown. ^**^The differences in NPQ values between the wild type plants (−) and mutants (+) are significant at *P* < 0.01.

To verify the function of *OsPsbS1 in vivo*, we first utilized RNA interference to silence the expression of *OsPsbS1*. The 425-bp fragment from the exon of *OsPsbS1* was cloned into RNAi constructs ds1301, which were then transformed into Zhonghua 11, a *japonica* variety. Further real-time PCR analysis showed that the transcription of *OsPsbS1* was drastically reduced in all transformants with much lower NPQ values (Figures 3A,B).

In the sequenced rice genome, *OsPsbS2*, another chlorophyll A-B binding protein gene, is found to be most similar to *OsPsbS1* with a nucleotide identity of 91%. To distinguish the function of these two homologous genes, we then used the CRISPR-Cas9 system with constructs containing Cas9 and sgRNA and targeting the single gene *OsPsbS1* or *OsPsbS2* and both of the two genes. The constructs were transformed into a *japonica* variety Zhonghua 11. Two target special sequences were designed for each gene. The potential mutations were detected by sequencing the PCR amplification products containing the two targets regions. Indels mutations were confirmed in the target regions. Compared with the wild-type plants, the *OsPsbS1* mutants and the double mutants exhibited much lower NPQ values (Figure 3C). However, no significant differences were found in NPQ values between the *OsPsbS2* mutants and the wild-type plants (Figure 3C).

**Figure 4:**
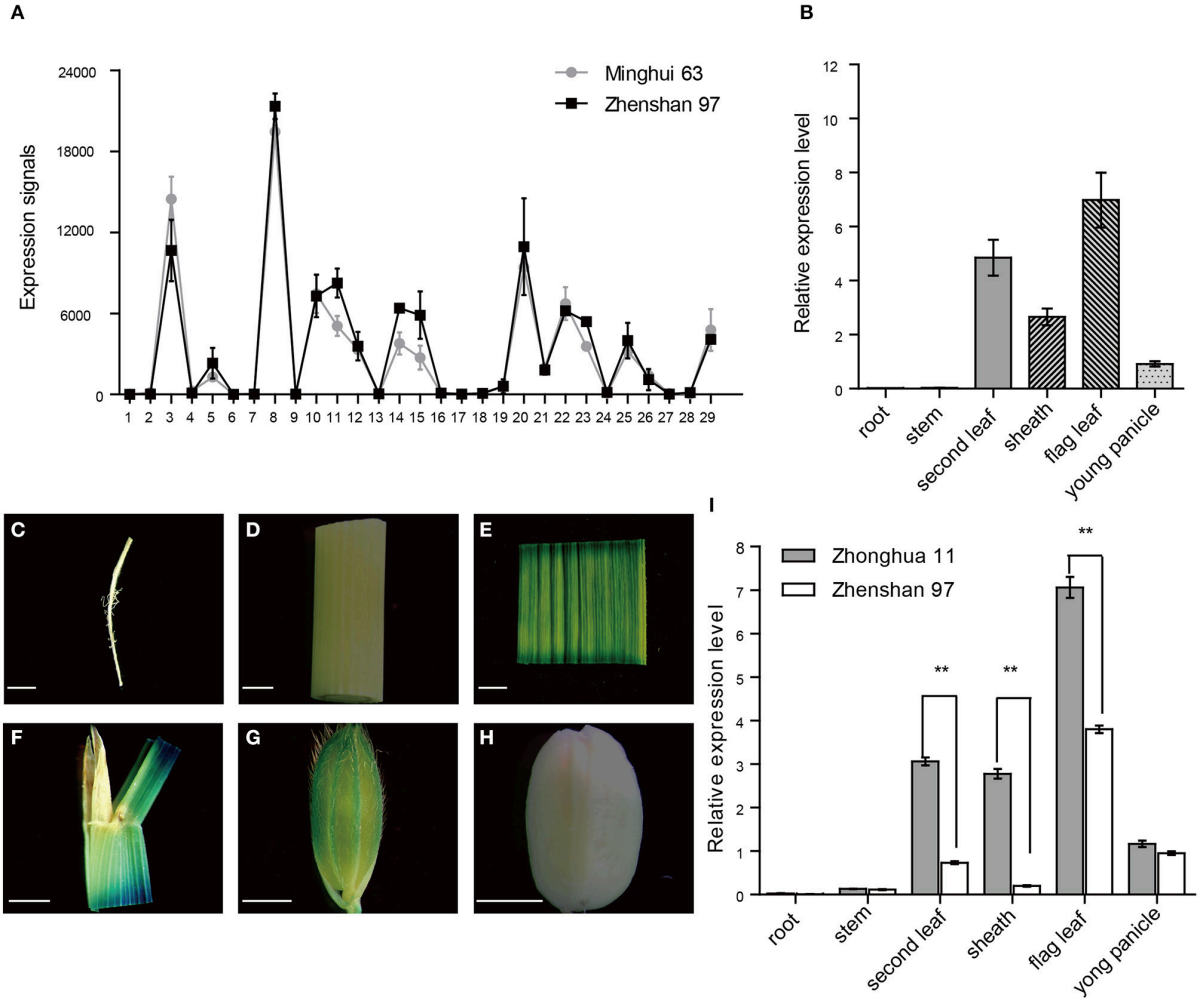
Expression patterns of *OsPsbS1*. **(A)** Expression signals of *OsPsbS1* in various tissues of Minghui 63 and Zhenshan 97 based on the microarray data. The X-axis represents the developmental stages which are listed in Table S1. The Y-axis represents the expression signals. **(B)** Real-time PCR analysis of *OsPsbS1* in various tissues of 02428. **(C–H)** *P*_*OsPsbS*1_*::GUS* analysis in various tissues of Zhonghua 11. GUS staining shown in primary root **(C)**, developing culm **(D)**, flag leaves **(E)**, leaf sheath **(F)**, young glume **(G)**, and mature seed **(H)**. Scale bars = 2 mm. **(I)** Comparison of the expression levels of *OsPsbS1* between Zhonghua 11 and Zhenshan 97 with different genotypes in the *OsPsbS1* promoter region by Real-time PCR. ^**^The differences in expression levels between Zhonghua 11 and Zhenshan 97 are significant at *P* < 0.01.

To get an overview of the expression profile of *OsPsbS1*, the CREP database (http://crep.ncpgr.cn/crep-cgi/home.pl), a website containing the dynamic gene expression atlas of *indica* rice, was searched (Wang et al., 2010). It was found that *OsPsbS1* exhibited high-level expression in leaf but low-level expression in root and stem in Minghui 63 and Zhenshan 97, two *indica* cultivars (Figure 4A). In addition, we also investigated the relative expression level of *OsPsbS1* in 02428, a *japonica* variety, by quantitative RT-PCR. Similarly, it was highly expressed in green tissues including leaf and leaf sheath (Figure 4B), indicating *OsPsbS1* was a green tissue-specific expressed gene. To complement the results at the mRNA level, a 3141-bp promoter region of *OsPsbS1* was ligated in frame with the β-glucuronidase (GUS) reporter gene, which was then introduced into Zhonghua 11, a *japonica* variety. GUS staining of the *P*_*OsPsbS*1_*::GUS* transgenic plants showed that *OsPsbS1* had specific and high-level expression in mature leaf (Figure 4E), leaf sheath (Figure 4F), and young glume (Figure 4G), which was in accordance with its high expression in the green tissues of 02428. No staining signals was observed in root (Figure 4C), stem (Figure 4D), and mature seed (Figure 4H). We further compared the expression levels of *OsPsbS1* in various tissues before heading stage between two varieties with different genotypes in the *OsPsbS1* promoter region by quantitative RT-PCR (Figure 4I). Zhonghua 11 contains the 2,674-bp insertion fragment in the promoter region, whereas Zhenshan 97 doesn't. The results showed that the expression level of *OsPsbS1* in Zhonghua 11 was much higher than that in Zhenshan 97 in green tissues such as leaves and shealth (Figure 4I). However, *OsPsbS1* exhibited very low-level expression in root and stem in both Zhonghua 11 and Zhenshan 97 (Figure 4I). In conclusion, *OsPsbS1* had specific and high-level expression in green tissues of rice, and genotypes with the 2674-bp insertion fragment in the promoter region tend to exhibit much higher level expression.

### Analysis of one candidate gene

**Figure 5:**
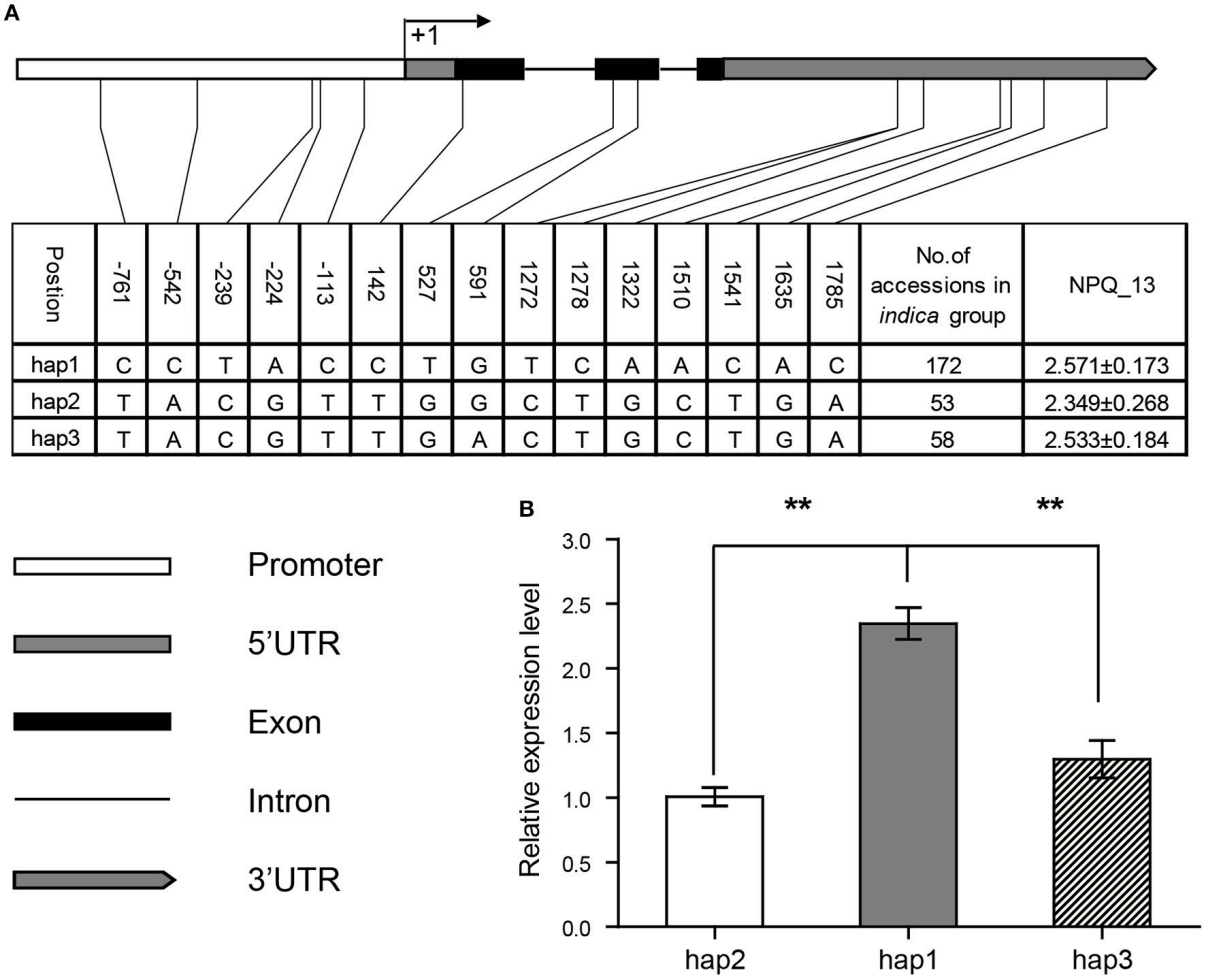
Haplotypes **(A)** and expression levels **(B)** of the candidate gene (LOC_Os01g01340) in *indica* group. Three major haplotypes were detected, and the number of accessions in each haplotype is shown. The positions of SNPs are shown in the first row. Trait value (NPQ_13) is indicated in the right column. Graphical representations of the gene structure and expression levels of the gene in different haplotypes are shown below. ^**^Indicates the differences of expression levels between hap1 and hap2 or hap3 are significant at *P* < 0.01.

A candidate gene encoding light-induced protein 1-like (LOC_Os01g01340) was located in one significant association region and close to the representative GWAS peak signal (around 129,845 on chromosome 1) for NPQ_13 in *indica* group (Table 2). Using resequencing data, the haplotypes of LOC_Os01g01340 were built based on SNPs and InDels detected in the promoter region (around 1 kb upstream of transcription initiation site), 5′ UTR, 3′ UTR, and non-synonymous SNPs and InDels in the coding region. We only focused on those haplotypes shared by at least 5 accessions in the analyzed group. Five haplotypes were found in the whole association panel (Figure S1). In total, five SNPs in the promoter region, three non-synonymous SNPs in the coding region, and seven SNPs in 3′ UTR were detected. Hap1, hap2, and hap3 were the three major haplotypes in *indica* group, whereas hap1 was the only major haplotype in *japonica* group (Figure S1). Considering the complexity of population structure and genetic background, we performed statistical analysis in the same or similar groups. Comparisons of the mean values of NPQ_13 showed that hap2 had significantly lower value than hap1 (*P* = 4.479 × 10^−12^) and hap3 (*P* = 1.227 × 10^−6^) in *indica* group (Figure 5). In addition, hap2 also exhibited significantly lower value than hap1 (*P* = 0.0019) and hap3 (*P* = 0.0260) in NPQ_12 in *indica*. We checked the expression of LOC_Os01g01340 in 59 accessions randomly chosen from *indica* with different haplotypes. Using qRT-PCR analysis, it was found that expression levels of LOC_Os01g01340 in hap1 accessions were much higher than those in hap2 and hap3 accessions (*P* < 0.01) (Figure 5, Table S5). These results indicate that this gene might be a good candidate for the GWAS locus.

## Discussion

The qE-type of NPQ is a very important photoprotective strategy for rice to adapt to the highly excessive natural illumination during summer growing season as well as the very dynamic light microenvironments inside canopy, which may be influenced minute by minute by wind, cloud, and time of day. In present study, using a diverse worldwide collection of 529 *O. sativa* accessions as the GWAS platform, we detected a total of 33 significant association loci for qE capacity. The validity of the GWAS signals was further demonstrated with three linkage mapping populations. All significant QTLs detected in bi-parental linkage mapping population could correspond to at least one GWAS signal. Compared with the intervals QTL mapping approach, our GWAS provided much higher resolution, facilitating candidate gene identification.

In this study, only three significant association regions were commonly detected by GWAS in two different groups. Although a total of 12, 6, and 4 significant association regions at LMM model were identified in *indica, japonica*, and *aus* subpopulations, respectively, there were no common QTLs detected between either of the two different subpopulations. These results indicate significant genetic heterogeneity of NPQ factors in different groups. We speculate that some of these QTLs might be fixed for one major haplotype in the subpopulation that failed in detection. For example, *OsPsbS1* can only be detected in the whole population but not in each subpopulation, with the main reason lying in that the major haplotype (presence or absence of the 2,674-bp insertion in the promoter region) was fixed and few polymorphisms were present in each subpopulation. In addition, consistent with the previous report by Demming-Adams and Adams (1994), we also observed that NPQ values were sensitive to environmental changes. Although the phenotyping was conducted for two years on an identical set of 529 *O. sativa* accessions under very similar cultivation conditions, only two regions could be repeatedly detected by GWAS in both 2012 and 2013. NPQ values in 2013 were significant higher than that in 2012, which might be due to the hotter and less cloudy days during the 2013 summer season.

Our GWAS clearly reveals *OsPsbS1* as a major determinant for natural variation of nonphotochemical quenching capacity in rice. *OsPsbS1* was repeatedly detected and explained more than 40% of the variation in the whole association population in both 2012 and 2013, and demonstrated to be a common major QTL in all three F2 mapping populations derived from inter-group crosses. It has been reported that, unlike typical light-harvesting complex proteins with three transmembrane helices, the PsbS protein has four helices that span the thylakoid membrane. Protein sequence analysis showed high similarity between helix I and helix III and also between helix II and helix IV. The pH-sensing mechanism of the PsbS protein in *Arabidopsis* is influenced by two pairs of symmetrically arranged glutamate residues, Glu-122 and Glu-226, each located within or close to the two lumen-exposed loops of the protein (Li et al., 2000, 2004). In this study, by sequencing 480 accessions, we found no nucleotide polymorphic sites for *OsPsbS1* in exon-1, intron-1, and exon-2, and only one synonymous SNP in exon-3, indicating the structure and function of PsbS1 protein are highly conserved in rice. In *Arabidopsis* mutants lacking PsbS are specifically defective in qE and more sensitive to photoinhibition (Li et al., 2002), and show decreased fitness under fluctuating light conditions in the field (Külheim et al., 2002). Consistent with the reports in *Arabidopsis*, we also observed CRISPR mutants for *OsPsbS1* exhibited drastically decreased NPQ values, and set very few seeds in the field. These findings suggest that the photoprotective processes conferred by qE serve fundamental roles in maintaining survival, reproduction, and fitness in plants.

In rice, there is another *PsbS* homologue, *OsPsbS2*. In present study, the whole genomic DNA sequences around *OsPsbS2* from 63 accessions in the association panel were also sequenced and analyzed (Table S2). Three synonymous SNPs and two non-synonymous SNPs were found in the exon. The putative transmembrane regions and glutamate residues are also conserved in PsbS2. The expression patterns were very similar to *OsPsbS1* (Figure S2). However, we didn't find significant function for *OsPsbS2* in its CRISPR mutants under the same conditions as *OsPsbS1* mutants, although there might be an additive effect in the double mutants when both *OsPsbS1* and *OsPsbS2* were knocked out (Figure 3C).

Due to the complexity of nature, identification of candidate genes for GWAS loci is generally difficult. The molecular mechanism underlying NPQ has been extensively investigated in Arabidopsis (Niyogi et al., 1998, 2001; Müller et al., 2001; Ruban, 2016). It is likely that the detected GWAS loci are rice homologues to the NPQ regulators identified in Arabidopsis. In addition to *OsPsbS1* and *OsPsbS2*, we also compared positions of the significant GWAS signals with xanthophyll cycle related rice homologues which include *OsVDE* (LOC_Os04g31040) and *OsZEP* (LOC_Os04g37619), lutein synthesis related homologues which include *OsLUT1A* (LOC_Os10g39930), *OsLUT1B* (LOC_Os02g57290), *OsLUT1C* (LOC_Os02g07680), and *OsLUT2* (LOC_Os01g39960), and cyclic electron flow related homologues which include *OsPGR5* (LOC_Os08g45190), *OsPGRL1A* (LOC_Os08g41460), and *OsPGRL1B* (LOC_Os03g64020) (DalCorso et al., 2008; Kasajima et al., 2011). However, except *OsPsbS1*, none of these rice homologues were found to correspond to the detected GWAS loci. Close to the GWAS peak signals, we did find some candidate genes (Table 2) that might be involved in processes of electron transport, buildup of proton gradient, PSII light harvesting antenna structure and light harvesting complex II (LHCII) rearrangements that trigger and regulate NPQ scenario (Ruban, 2016). Considering the strong population structure and complicated genetic background, we further chose a candidate gene (LOC_Os01g01340) close to one representative GWAS peak signal for NPQ_13 in *indica* group and analyzed in depth. The results support that it might be a good candidate for the GWAS locus. Before our study, the function of this gene hasn't been reported. In addition, it is interesting to note that, *Grain number, plant height, and heading date7* **(***Ghd7*), a gene with pleiotropic effects on plant height, heading date, yield traits, and chlorophyll content (Xue et al., 2008; Wang et al., 2015), is in the very close proximity of one representative GWAS peak signal (around 9,162,584 on chromosome 7) that repeatedly detected in both NPQ_12 and NPQ_13. We thus suggest it as candidate gene for the GWAS locus (Table 2).

Manipulating photoprotective mechanism has been suggested and demonstrated to be an effective means to enhance both stress resistance and photosynthetic productivity of crop plants (Horton, 2000; Zhu et al., 2010; Murchie and Niyogi, 2011; Kromdijk et al., 2016). This study unraveled that *OsPsbS1* is a major determinant for natural variation of qE capacity. In addition, we revealed dozens of significant association loci, most of which explained more than 5.0% of the phenotypic variance. In future, QTLs accelerating the recovery of NPQ should also be identified. Systematic combination and manipulation of these QTLs may increase qE capacity, enhance photoprotection, and improve rice photosynthesis and yield in adverse environments.

## Conclusions

Thirty-three significant association loci for natural variation of NPQ capacity in rice were identified by GWAS, and the validity of the GWAS signals was demonstrated by linkage mapping in three F2 populations. Candidate genes underlying some significant association regions were proposed. *OsPsbS1* was found to be a major determinant for NPQ variation explaining more than 40% of the variation in the whole association population, and a common major QTL in all mapping populations derived from inter-group crosses. The nucleotide diversity analysis reveals no non-synonymous SNPs or InDels in the coding region, indicating the PsbS1 protein sequence is highly conserved. Haplotypes with the 2,674-bp insertion in the promoter region exhibited significantly higher NPQ values and higher expression levels of *OsPsbS1* in leaves. The *OsPsbS1* RNAi plants and CRISPR mutants exhibited drastically decreased NPQ values. *OsPsbS1* had specific and high-level expression in green tissues. A candidate gene encoding light-induced protein 1-like (LOC_Os01g01340) was also analyzed. The significant loci detected in present study would help enhance photoprotection and improve photosynthesis in rice.

## Author contributions

GW designed the study and analyzed the data. QW carried out most of the experiments and analyzed the data. HZ and WX performed GWAS. Other authors assisted in experiments. GW and QW wrote the manuscript. JJ and JX performed genotypic assay in QTL mapping. XF and CL measured NPQ values. YH assisted in the construction of mapping populations.

### Conflict of interest statement

The authors declare that the research was conducted in the absence of any commercial or financial relationships that could be construed as a potential conflict of interest.
