# Supplementary material for: Genetic Architecture of Natural Variation in Rice Nonphotochemical Quenching Capacity Revealed by Genome-Wide Association Study
Source: Front Plant Sci. 2017 Oct 13;8:1773. doi: 10.3389/fpls.2017.01773 (PMC5645755; doi:10.3389/fpls.2017.01773)
Supplement: Supplementary file 1 [file DataSheet1.DOCX]

**SUPPLEMENTARY MATERIAL**

**Table S1 Detailed information of rice samples used in microarray**

| Number | Developmental stages analyed |
| --- | --- |
| 1 | Seed: germination (72 h after imbibition) |
| 2 | Calli: 15 day after induction |
| 3 | Embryo and radicle after germination |
| 4 | Plumule 1: 48h after emergence, dark |
| 5 | Plumule 2: 48h after emergence, light |
| 6 | Radicle 1: 48h after emergence, dark |
| 7 | Radicle 2: 48h after emergence, light |
| 8 | Leaf and root at three-leaf stage |
| 9 | Root: seedling with 2 tillers |
| 10 | Shoot: seedling with 2 tillers |
| 11 | Leaf 1: secondary branch primordium differentiation stage |
| 12 | Sheath 1: secondary branch primordium differentiation stage |
| 13 | Panicle 1: secondary branch primordium differentiation stage |
| 14 | Leaf 2: 4-5cm young panicle |
| 15 | Sheath 2: 4-5cm young panicle |
| 16 | Panicle 2: 4-5cm young panicle |
| 17 | Panicle 3: pistil/stamen primordium differentiation stage |
| 18 | Panicle 4: pollen-mother cell formation stage |
| 19 | Culm 1: 5 days before heading |
| 20 | Flag leaf 3: 5 days before heading |
| 21 | Culm 2: heading stage |
| 22 | Panicle 5: heading stage |
| 23 | Glume: one day before flowering |
| 24 | Stamen: one day before flowering |
| 25 | Spikelet: 3 days after pollination |
| 26 | Endosperm 1: 7 days after pollination |
| 27 | Endosperm 2: 21 days after pollination |
| 28 | Endosperm 3: 14 days after pollination |
| 29 | Flag leaf 4: 14 days after heading |

**Table S2 Summary of DNA polymorphic sites of *OsPsbS2* genome**

| Parameter | Entire region | 5’UTR | Exon1 | 3’UTR |
| --- | --- | --- | --- | --- |
| Length, bp | 1126 | 145 | 765 | 216 |
| SNP sites | 6 | 1 | 5 | 0 |
| Whole population |  |  |  |  |
| π | 0.00241 | 0.00433 | 0.00280 | 0 |
| θ | 0.00116 | 0.00181 | 0.00139 | 0 |
| Tajima’s *D* | 2.62283* | 1.76166 | 2.34851* | - |

π, average number of nucleotide differences per site between two sequences; θ, Watterson estimator; Tajima’s *D*, test for neutral selection. *Significant at *P*<0.05

**Table S3 Primers used in this study**

| Primer name | Primer sequence(5'-3') |
| --- | --- |
| **Primers for *OsPsbS1* PCR amplification and sequencing** | |
| PsbS1-0-F | GGTGGTGGCGTTGTCTTTAG |
| PsbS1-R | GTGTGACTCTGCGTGTTGTG |
| PsbS1-M-0-F | ATGGCATTGTGTGCCATC |
| PsbS1-1-F | ATCTGGTGGCTTGGGGGTA |
| PsbS1-1-R | TGTATCAGCTCGCTTCCGT |
| PsbS1-M-R | GTGGACCTACCTTGTGTAC |
| PsbS1-2-F | ATCGGTATGCTGGAGATGTTG |
| PsbS1-2-R | TGCATATATTTCAGCAGGGCA |
| PsbS1-M-2-F | CTACAGGACAAGCAAATGG |
| PsbS1-M-2-R | TCATACAGTAACGCTGCTC |
| PsbS1-3-F | CCCAACTCAACTCATCGTC |
| PsbS1-3-R | GAGAGGGAGATCCTGTGGC |
| PsbS1-4-F | CAACCACCTGCTCTTCTCGG |
| PsbS1-4-F | TGTCGCTGTCAAAACTCGGA |
| PsbS1-5-F | AAGAGCACAAGCTTAAACGT |
| PsbS1-5-F | GATGAAGAAGAGGAGGAGGG |
| PsbS1-5-M-F | CAACCTAACGTTAATCCTGC |
| PsbS1-5-M-R | CATCACTTTGGTTCGACTC |
| PsbS1-6-F | ATACCAACCTGATGCAAAAGC |
| PsbS1-6-R | CGAATGTAACTTTAGCATGTC |
| **Primers for GUS staining** | |
| P1-GUS-0F | GCCCTTGCTCACCATGGATCC CCTGCTCTTCTCGGCCAGT |
| P1-GUS-0R | GACCACCCGGGATCTCTGCAGGGTCGCGGTGTGCGTACGT |
| P1-GUS-5F | GCCCTTGCTCACCATGGATCCCGTGGCACACAGTTCACTA |
| P1-GUS-5R | GACCACCCGGGATCTCTGCAGGGTCGCGGTGTGCGTACGT |
| **Primers for cloning** | |
| P1-RNAi-F | AACTAGTGGTACCATCCCGATCTACGAGGCGGAG |
| P1-RNAi-R | AGAGCTCGGATCCTACTCTTCGTCGTCGTCGCTG |
| CRISPR for targeting *OsPsbS1* | |
| P1 -U3-F | GGCATGCTGGTGTCGGGAGCCAA |
| P1 -U3-R | AAACTTGGCTCCCGACACCAGCA |
| P1 -U6a-F | GCCGAAGCCAAAGTTCAAGACCG |
| P1-U6a-R | AAACCGGTCTTGAACTTTGGCTT |
| CRISPR for targeting *OsPsbS2* | |
| P2-U3-F | GGCAATGGCTCTGCAGCAGAGCA |
| P2-U3-R | AAACTGCTCTGCTGCAGAGCCAT |
| P2-U6a-F | GCCGATGATGGTAGTGTCTGACCT |
| P2-U6a-R | AAACAGGTCAGACACTACCATCAT |
| CRISPR for targeting both *OsPsbS1* and *OsPsbS2* | |
| P1 -U3-F | GGCATGCTGGTGTCGGGAGCCAA |
| P1 -U3-R | AAACTTGGCTCCCGACACCAGCA |
| P1 -U6a-F | GCCGAAGCCAAAGTTCAAGACCG |
| P1-U6a-R | AAACCGGTCTTGAACTTTGGCTT |
| P2-U6b-F | GTTGAACAAGAACAAGTCGATCG |
| P2-U6b-R | AAACCGATCGACTTGTTCTTGTT |
| P2-U6c-F | TCAGAAGAAGCATCTGGTGGTGG |
| P2-U6c-R | AAACCCACCACCAGATGCTTCTT |
| Primers for CRISPR sequencing | |
| cas9-F | GGTCGCCTACCACGAGAAGTACC |
| cas9-R | GTGAGGTCCTGGTGGTGCTCGTC |
| P1-seq-F | CTCATAAAAACCTCGCCACCT |
| P1-seq-R | AGGTCTACTTACGGCAAACCC |
| P2-seq-F | TCCAAGAGAGCAAGCCAAGAT |
| P2-seq-R | AGGTTGAGCTGCGCCAGGAT |
| **Primers for Real-time PCR** | |
| UBIQ-F | AACCAGCTGAGGCCCAAGA |
| UBIQ-F | ACGATTGATTTAACCAGTCCATGA |
| RT-PsbS1-F | CTGTTCGGCAGGTCCAA |
| RT-PsbS1-R | ACGAACAGCTCGTTCTCCT |
| RT-01g01340-F | GCTGCGGAGGTGGACTACAG |
| RT-01g01340-R | CTGGGTACATCTGCACGTTG |

**Table S4 SSR markers used in the QTL analysis (xls)**

**Table S5 Relative expression of LOC_Os01g01340 in 59 accessions randomly chosen from *indica* with different haplotypes (xls)**

**
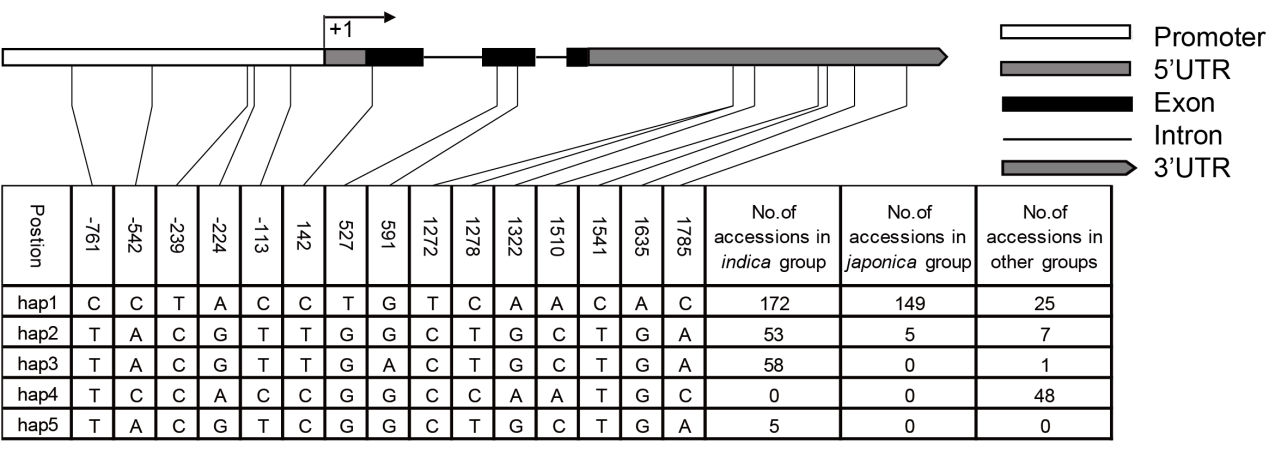
Figure S1 Haplotypes of the gene (LOC_Os01g01340) and their distribution in groups of the whole association panel**

Five haplotypes were detected, and the number of accessions of each haplotype in groups is shown. The positions of SNPs are shown in the first row. Graphical representations of the gene structure are indicated.


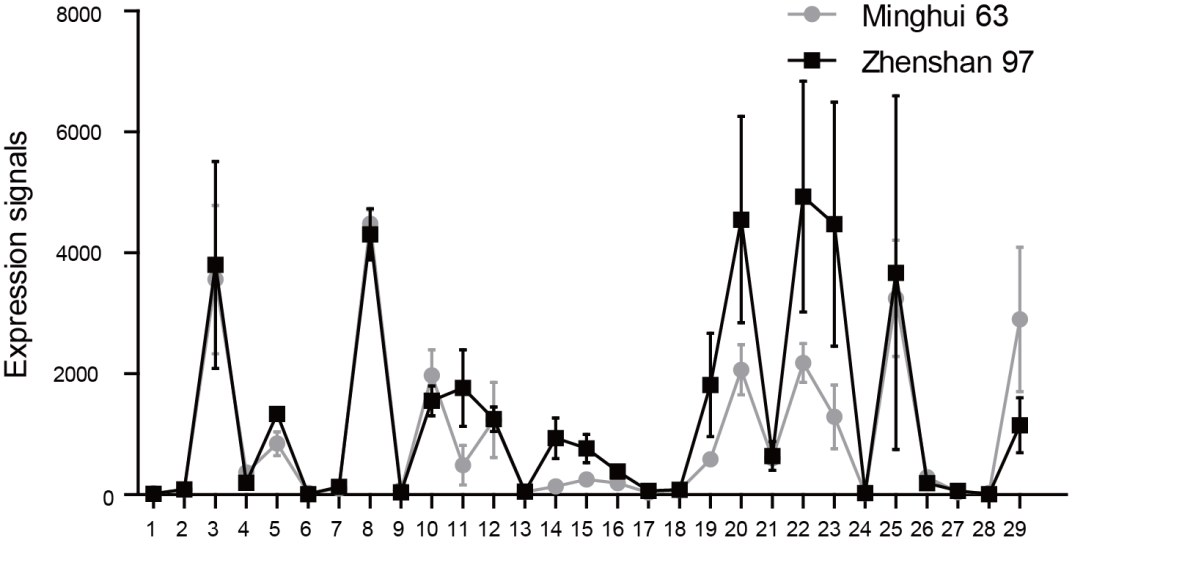


**Figure S2 Expression patterns of *OsPsbS2***

The expression signals of *OsPsbS2* in various tissues of Minghui 63 and Zhenshan97 were based on the microarray data. The X-axis represents the developmental stages which are listed in Table S1.
